# Unraveling the association between genetic integrity and metabolic activity in pre-implantation stage embryos

**DOI:** 10.1038/srep37291

**Published:** 2016-11-17

**Authors:** Fiona D’Souza, Shivanand M. Pudakalakatti, Shubhashree Uppangala, Sachin Honguntikar, Sujith Raj Salian, Guruprasad Kalthur, Renu Pasricha, Divya Appajigowda, Hanudatta S. Atreya, Satish Kumar Adiga

**Affiliations:** 1 Division of Clinical Embryology, Centre of Excellence in Clinical Embryology, Kasturba Medical College, Manipal University, Manipal, India; 2 NMR Research Centre Indian Institute of Science, Bengaluru, India; 3 Solid State and Structural Chemistry Unit, Indian Institute of Science, Bengaluru, India; 4 National Centre For Biological Sciences, TIFR, Bengaluru, India

## Abstract

Early development of certain mammalian embryos is protected by complex checkpoint systems to maintain the genomic integrity. Several metabolic pathways are modulated in response to genetic insults in mammalian cells. The present study investigated the relationship between the genetic integrity, embryo metabolites and developmental competence in preimplantation stage mouse embryos with the aim to identify early biomarkers which can predict embryonic genetic integrity using spent medium profiling by NMR spectroscopy. Embryos carrying induced DNA lesions (IDL) developed normally for the first 2.5 days, but began to exhibit a developmental delay at embryonic day 3.5(E3.5) though they were morphologically indistinguishable from control embryos. Analysis of metabolites in the spent medium on E3.5 revealed a significant association between pyruvate, lactate, glucose, proline, lysine, alanine, valine, isoleucine and thymine and the extent of genetic instability observed in the embryos on E4.5. Further analysis revealed an association of apoptosis and micronuclei frequency with P53 and Bax transcripts in IDL embryos on the E4.5 owing to delayed induction of chromosome instability. We conclude that estimation of metabolites on E3.5 in spent medium may serve as a biomarker to predict the genetic integrity in pre-implantation stage embryos which opens up new avenues to improve outcomes in clinical IVF programs.

Genotoxic stress is known to severely compromise genomic integrity. Majority of the cell types instantly activate cell cycle checkpoint systems when replication is stalled by DNA damage^1^. Failure to repair DNA lesions completely before the induction of cell proliferation can lead to genomic instability. As such events may have serious health implications, damaged cells are often eliminated via apoptosis as a fail-safe mechanism^2^,^3^.

Preimplantation stage embryos are sensitive to genotoxic agents such as radiation^4^ and chemotherapeutic agents^5^ and this could be owing to a peculiarity of the damage responses of the early-stage embryos^6^,^7^. Apart from species specific difference, cell cycle regulations also vary between somatic and embryonic cells within a species. However, Drosophila, Zebrafish and *Xenopus* embryos carrying DNA lesions failed to arrest even when DNA synthesis was inhibited by aphidicolin^8^,^9^,^10^. Similarly, about half of the human embryos derived *in vitro* are known to possess chromosomal abnormalities even while being developmentally and morphologically indistinguishable from euploid embryos^11^. This is mainly due to the fact that human embryos in the preimplantation stage are prone to genomic errors thus acquire increased incidence of DNA abnormalities which is further propagated by the increased expression of cell cycle drivers and inadequately expressed cell cycle check point regulators^12^,^13^. Adding to this is the inability of morphology based conventional embryo selection criteria practiced in assisted reproduction techniques to ascertain the genetic health of the embryo. This eventually raises the risk of abnormal reproductive outcome when such embryos are mistaken to be healthy and are transferred during *in vitro* fertilization procedures.

The unique stage specific metabolic requirement and the ability of preimplantation stage embryos to modify their immediate environment makes the study of embryonic metabolism instrumental in depicting the intrinsic state of the embryo non-invasively. Healthy embryos are metabolically quiescent, thus it is suggested that any pathological condition that compromises the quality of the embryo elicits an active metabolic response which can be detected as increased turnover of amino acids and energy substrates from the embryo culture medium^14^. A recent study has shown that changing the metabolite concentration affects cell phenotypes in the embryo^15^. A number of studies have attempted to non-invasively measure embryo quality based on the metabolic signatures of the embryo culture media by using a variety of techniques^16^,^17^,^18^,^19^,^20^. Nonetheless, the extension of these findings to clinical application remains clouded by conflicting reports^21^ and technical complexities. Our group has demonstrated that uptake of pyruvate by the human embryos from culture medium and pyruvate to alanine ratio on day 3 of development is predictive of implantation potential^20^. In the present study, we investigated the relationship between the genetic integrity, embryo metabolism and developmental competence in preimplantation stage mouse embryos with the aim to identify early biomarkers which can predict embryonic genetic integrity using spent medium profiling by nuclear magnetic resonance (NMR) spectroscopy.

## Results

### Impaired developmental competence due to induced DNA lesions at post compaction phase

**Figure 1:**
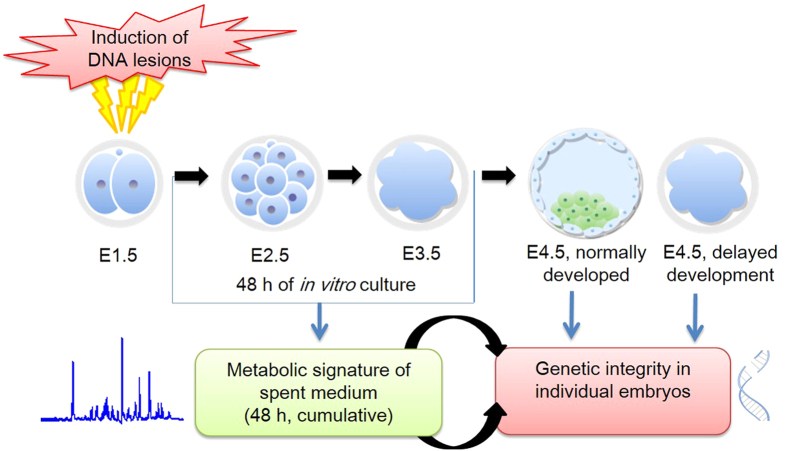
Overview of the experimental design for data depicted in the study.

**Figure 2:**
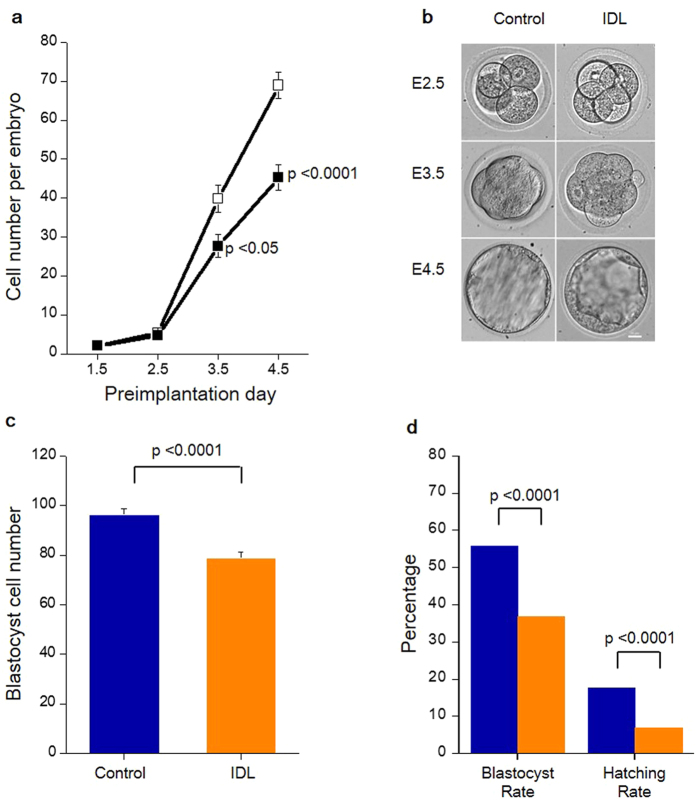
Preimplantation development of IDL embryos (**a**) Total cell number in control (□) and IDL (■) embryos at different developmental stages. Embryos were stained with DAPI to determine total cell number at each day of development. Control E1.5 (N = 67), E2.5 (N = 67), E3.4 (N = 34) and E4.5 (N = 61). IDL group E1.5 (N = 81), E2.5 (N = 81), E3.4 (N = 36) and E4.5 (N = 81). Total cell number in IDL embryos was significantly affected on E3.5 (p < 0.05) and E4.5 (p < 0.0001). Difference between control and test group was evaluated using Student’s t-test. (**b**) Morphology of control and IDL embryos at different developmental stages. Scale bar = 25 μm (**c**) Blastocyst cell number on E4.5. The number of cell in embryos that successfully developed to the blastocyst stage was significantly reduced (p < 0.0001) in the IDL group vs the control group. (**d**) Developmental potential of IDL embryos on E4.5. Blastocyst rate was significantly (p < 0.0001) reduced in IDL group (N = 322/877) when compared to the control (N = 271/485). Further, hatching rate was significantly (p < 0.0001) affected in IDL group (N = 322/877) compared to the control (N = 271/485). Data represented as percentage derived from a total of 17 experiments. Blue bars represent control and orange bars represent IDL group. Statistical significance between the two groups was evaluated by Chi-square test.

To understand the ability of preimplantation embryos to modulate their immediate environment while developing into morphologically healthy blastocysts in spite of carrying underlying DNA lesions, DNA damage in 2 cell stage embryos was induced by exposure to 3 μM Cisplatin. Cisplatin is an antineoplastic agent which is known to act by the induction of DNA intra- and inter-strand cross-links^22^. Figure 1 represents the overview of the experimental design for data depicted in the study. The developmental potential in terms of total cell number and morphology of the embryos carrying induced DNA lesions (hereafter referred as IDL embryos) is plotted in Fig. 2a,b, did not show any evidence of developmental delay until day 2.5 of development (hereafter referred as E2.5). However, a significant (p < 0.05) delay in cell progression was evident from E3.5 when the embryos began to compact (Fig. 2a). Total cell number, blastocyst rate and hatching potential in IDL embryos were significantly affected on E4.5 (p < 0.0001) (Fig. 2a,d). However, the blastocysts derived from IDL group on E4.5 were morphologically indistinguishable from control blastocysts (Fig. 2b). Interestingly, comparison of total cell number in morphologically normal blastocysts on E4.5 was made in both control and IDL group revealed that IDL blastocysts had significantly lower number of cells compared to control group (p < 0.0001, Fig. 2c).

### Delayed activation of apoptosis and micronuclei in IDL embryos are associated with the expression of apoptotic regulators

**Figure 3:**
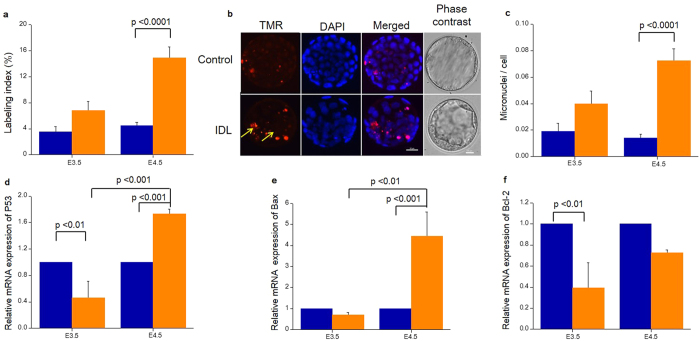
Delayed and stage specific response to the induction of DNA lesions on E1.5. (**a**) TUNEL assay was performed on E3.5 and E4.5 embryos. Incidence of labelling index (LI) was significantly higher on E4.5, control vs IDL group (p < 0.0001). (**b**) Representative image of TUNEL assay on E4.5. The first panel represents embryos stained with TMR Red in which red signals indicate TUNEL positive cells. The second panel is those stained with DAPI, the third panel is merged images and the fourth panel represents the phase contrast image prior to fixation for TUNEL assay. Scale bar = 25 μm. (**c**) Genetic instability in IDL embryos manifested as micronuclei on E3.5 and E4.5. Incidence of micronuclei was significantly increased in IDL embryos on E4.5 (control vs IDL group, p < 0.0001). (**d**) Expression of P53 mRNA. P53 transcripts were down regulated in IDL embryos on E3.5 (control vs IDL p < 0.01) and up regulated on E4.5 (control vs IDL, p < 0.001, E3.5 vs E4.5 in IDL embryos, p < 0.001). (**e**) Expression of Bax mRNA. Bax transcripts were up regulated on E4.5 (control vs IDL, p < 0.001, E3.5 vs 4.5 in IDL embryos, p < 0.01). (**f**) Expression of Bcl-2 mRNA. Bcl-2 transcripts were downregulated on E3.5 (control vs IDL embryos, p < 0.01) and E4.5 (NS). Statistical significance evaluated using Student’s t-test. Blue bars represent control and orange bars represent IDL group. Data represented as Mean ± Standard error of the mean (SEM).

**Figure 4:**
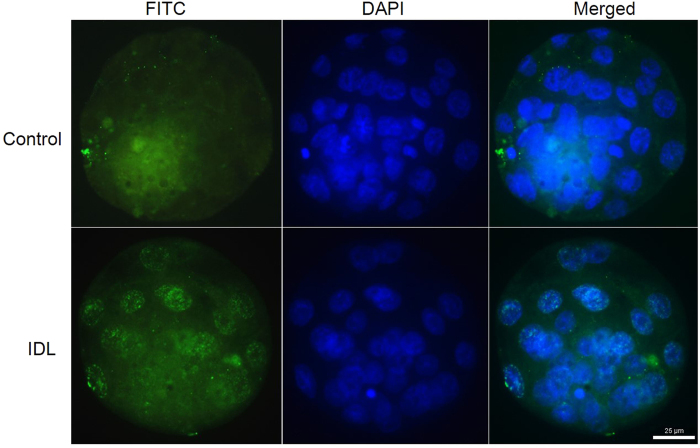
Representative image showing γH2AX foci demonstrating the DNA double strand breaks in IDL embryos on E4.5. The first panel represents embryos stained with γH2AX (FITC) in which green signals indicate DNA DSB’s. The second panel is those stained with DAPI, the third panel is merged images. Scale bar = 25 μm.

**Figure 5:**
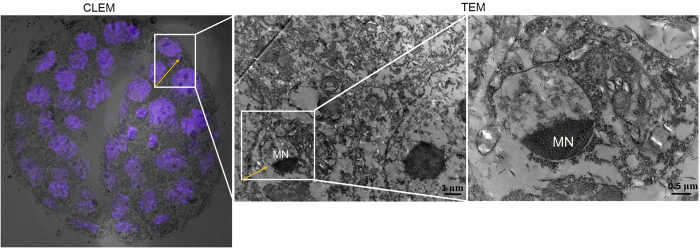
Correlative Light and Electron Microscopy (CLEM) to ascertain the presence of a nuclear membrane around the micronucleus. The CLEM image showing the embryo stained with DAPI. and the zoomed ROI (region of interest) images of TEM.

The incidence of apoptosis as determined by labeling index (LI) in embryos on E3.5 was not significantly different between the control and IDL embryos (3.51 ± 0.83 vs 6.8 ± 1.39). In contrast, approximately a three-fold increase in LI was observed in IDL embryos in comparison to control on E4.5 (p < 0.0001) (Fig. 3a,b). The compromised genetic integrity in these embryos did not manifest into morphologically visible traits such as degeneration or fragmentation (Fig. 3b). H2AX histone characteristically undergoes phosphorylation at serine 139 in response to DNA damage and DNA double strand breaks (DSBs). Immunolocalization of γH2AX in IDL embryos showed distinct foci confirming the presence of DNA DSBs in IDL embryos (Fig. 4). Genetic insult-induced DNA double-strand breaks produce chromosome breaks which can be detected as micronuclei (MN) (Fig. 5). Indeed, DNA double-strand breaks were evident as the foci of γH2AX overlapped with MN in embryos. Since the DNA structures in the micronucleus could represent condensed chromatin preparing for cell division and DAPI staining of DNA alone is not adequate to confirm the micronucleus structure, we applied Correlational Light/Fluorescent Transmission Electron Microscopy (CLEM) to ascertain the presence of a nuclear membrane around the micronuclei (Fig. 5). Analyses of IDL embryos on E4.5 for micronuclei frequency showed approximately 5-fold increase in micronuclei compared to control (p < 0.0001). In contrast, MN frequency on E3.5 did not significantly vary between IDL and control embryos (Fig. 3c). It is interesting to note that morphological parameters indicated that embryos with compromised genetic integrity were indistinguishable from the control group (Fig. 2b). Granulation or fragmentation that would implicate loss of viability was also not observed in IDL embryos.

Expression pattern of P53, Bax and Bcl-2 mRNA was assessed in IDL embryos on E3.5 and E4.5. P53 transcripts in IDL embryos were approximately 0.5 fold lower than the control in E3.5 embryos (p < 0.01). On the other hand, on E4.5, the expression level of P53 was increased by 1.76 fold in IDL embryos (p < 0.001) (Fig. 3d). Similarly, pro apoptotic regulator Bax also showed almost 4.5- fold increased expression in IDL embryos compared to control on E4.5 (p < 0.001, Fig. 3e). In contrast, anti-apoptotic Bcl-2 was poorly expressed in IDL embryos on E4.5 (Fig. 3f). It is important to note that GAPDH, the reference gene expression was not significantly different across the groups (Supplementary Figure S1).

### Modulation of metabolite levels in embryos

The metabolite levels in the medium across the different experimental categories, as quantified using NMR, were analyzed in two different ways. In one approach, we directly compared the relative metabolite levels in the spent medium on E3.5 between the embryos with normal and delayed growth in both control and IDL groups. This is shown in Tables 1 and 2 and discussed below. In the second approach, the apoptotic (labeling) index (LI) in individual embryos on E3.5 and E4.5 was compared with the metabolic profile of the spent embryo culture medium to assess the metabolic alterations induced by the presence of underlying DNA lesions.

### Uptake of metabolites in relation to the embryo developmental competence

**Figure 6:**
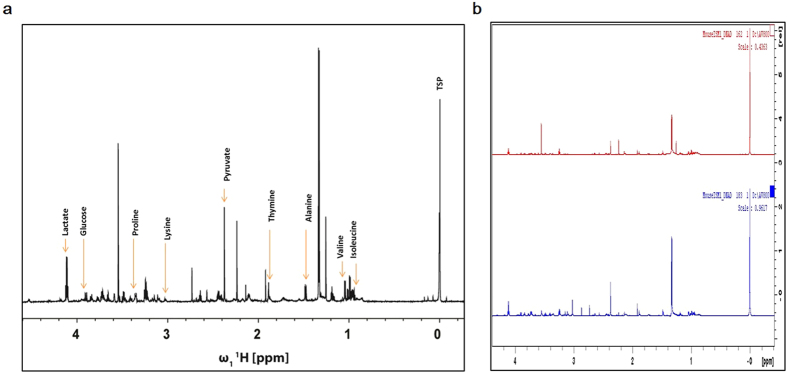
(**a**) 1D^1^ H NMR spectrum of ISM1 medium with chemical shift assignments of a few key metabolites indicated. (**b**) Representative spectrum from an IDL embryo carrying more than 12% LI (Red spectrum) and control embryo without apoptosis (Blue spectrum).

The cumulative metabolic activity depicted in 48 h culture medium of individual embryos was evaluated on E3.5. A total of 185 1D ^1^H NMR spectra were acquired from both IDL (N = 97) and control (N = 72) groups to understand the association between the metabolic signature and developmental potential of the embryos. In addition, 16 media samples were incubated in identical conditions without any embryos to determine the baseline concentration of metabolites. The characteristic NMR signals corresponding to components of ISM1 media were assigned^20^. Figure 6a,b shows the assignments of the metabolites in a representative 1D ^1^H spectrum. Among the metabolites present in ISM1 medium, the resonances of glucose, lactate, pyruvate, thymine and the following five amino acids: alanine, proline, valine, lysine and isoleucine were observed to be well resolved facilitating in their unambiguous quantitative assessment relative to the reference (TSP).

Table 1 shows the relative levels of the different metabolites across the different samples. Note that an increase in the uptake of metabolite results in its decrease in the medium. A significant increase in the uptake of glucose (p < 0.05), pyruvate (p < 0.01), alanine (p < 0.01), valine (p < 0.05), isoleucine (p < 0.05) and thymine (p < 0.01) was observed in the control embryos that developed to the blastocyst stage (Column 1 of the Table 1) when compared to embryos that were delayed at the compaction stage (Column 2 of the Table 1) on E3.5. A significant difference only in thymine (p < 0.05) was noted between normally developed and delayed IDL embryos on E3.5 (Column 3 and 4 of the Table 1). These observations suggest that metabolic uptake is related to the stage of the embryos in the absence of DNA damage (as shown in the previous study^20^).

Next, both normally developed and delayed embryos were further cultured to E4.5 to study the predictive value of E3.5 metabolic profiling in describing their developmental potential. The relative metabolites levels are shown in Table 2. The uptake of pyruvate (p < 0.01), valine (p < 0.05), and thymine (p < 0.05) on E3.5 was significantly higher in embryos that developed normally when compared to the delayed embryos on E4.5 for control embryos (Columns 1 and 2 of the Table 2). Additionally, blastocysts that underwent hatching on E4.5 had increased uptake of thymine (p < 0.01) on E3.5 when compared to unhatched blastocysts in the control group. However, as observed on E3.5 (Table 1) the metabolic signature of normally developed IDL embryos on E4.5 did not differ significantly from delayed embryos (Columns 3 and 4 of the Table 2). This is possibly due a wider range of LI observed in the IDL embryos compared to the control, which could be masking the differences between the normally developed and delayed IDL embryos, both of which are expected to have a broad range of LI. To unravel this, cells were segregated based on LI and the metabolite levels analyzed as described below.

### Genetic instability in E4.5 IDL embryos reflected in metabolites of E3.5 embryo spent culture medium

To further investigate the metabolic alterations induced by the presence of underlying DNA lesions, LI was calculated in individual embryos on E3.5 and E4.5 and related to the metabolic profile of the spent embryo culture medium. Though LI in IDL embryos on E3.5 did not show significant difference from control embryos, it was almost doubled on E4.5 in IDL embryos and the difference was statistically significant from control (p < 0.0001) (Fig. 3a). This observation is supported by the micronuclei (MN) frequency in IDL embryos which also demonstrated a significant increase from control (p < 0.0001) only on E4.5 (Fig. 3c).

We attempted to understand the association between genetic instability manifested as apoptosis in E4.5 embryos and the metabolic signature observed in embryo spent medium on E3.5. On analyzing the distribution pattern in LI among control and IDL embryos, it was evident that the LI observed in the control group was not higher than 12%. On the other hand, 38.3% of IDL embryos had LI > 12% on E4.5. Thus two subgroups were made in the IDL embryos based on LI (viz. <12% and >12%).

The MN frequency also showed a positive correlation with LI (R = 0.29, p < 0.05) where approximately 5- fold increase in MN was observed in IDL embryos with >12% LI. The metabolite intensities in the control and the IDL embryos with <12% LI was statistically insignificant which suggests that cisplatin independently did not modulate the metabolism in these embryos.

**Figure 7:**
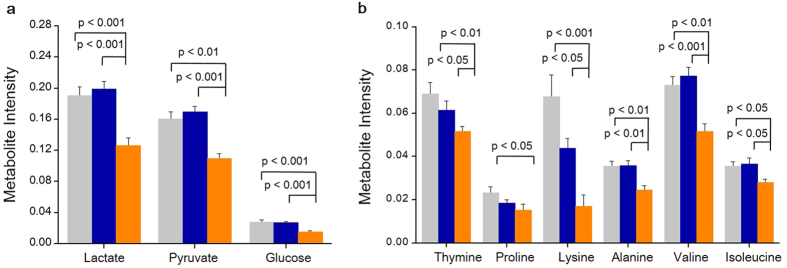
Metabolites from spent culture medium on E3.5 in relation to LI on E4.5. Light grey bars represent control (N = 19); blue bars represent IDL embryos with <12% LI (N = 15) and orange bars represent IDL embryos >12% LI (N = 15). Statistical significance evaluated using Student’s t-test. No significant differences were observed in between control and IDL embryos with <12% LI. Data is represented as Mean ± Standard error of the mean (SEM).

A strikingly altered metabolic pattern was observed between control group and IDL embryos with >12% LI. IDL embryos with >12% LI had a significantly higher requirement for the energy substrates lactate (p < 0.001), pyruvate (p < 0.01), glucose (p < 0.001) compared to control group (Fig. 7a). In addition, the uptake of amino acids such as proline (p < 0.05), lysine (p < 0.001), alanine (p < 0.01) valine (p < 0.01) and isoleucine (p < 0.05) was elevated in the embryos with >12% LI. Correlational analysis revealed that the uptake of lactate (p < 0.05), pyruvate (p < 0.001), glucose (p < 0.05), proline (p < 0.05), alanine (p < 0.05) and valine (p < 0.05) significantly correlated with increased LI (Fig. 7b). Additionally IDL embryos with >12% LI also had increased requirement for thymine (p < 0.01, Fig. 7b).

Further, we assessed the effectiveness of metabolomics vs morphology assessment in detecting the apoptosis on E4.5 blastocysts. The DNA lesions as measured by LI varied significantly (p < 0.001) across blastocysts from control and IDL groups on E4.5. The metabolite levels in both groups were assessed to understand the association with LI and metabolic uptake in morphologically normal embryos. Interestingly, the intensity of glucose (p < 0.05), thymine (p < 0.05), proline (p < 0.05) and lysine (p < 0.01) was significantly lower in IDL blastocysts carrying DNA lesions. These observations suggest that metabolomics may predict embryonic apoptosis non-invasively at least 24 h before where embryos were morphologically indistinguishable. From the above sets of experiments, it is clear that the metabolite levels in the medium vary in relation to morphology and development especially in control group and to a certain extent in IDL embryos on E3.5. It is important to note that a significant reduction in total cell number in IDL embryos (though embryos appeared morphologically normal) possibly affected lack of overall variation in metabolite levels on E4.5. Therefore, an additional experiment was conducted where the embryonic metabolism on E3.5 was normalized to the cell number in the embryos on the same day. No significant differences were observed in the metabolism between the groups (Supplementary Table S1). However, segregating IDL embryos based on LI revealed significant metabolic alterations induced by the presence of underlying DNA lesions.

## Discussion

This is the first approach to elucidate the metabolic response in mouse preimplantation embryos to experimentally induced DNA lesions. This approach provided valuable insights on changes in requirement of certain embryo metabolites whose levels were altered in the spent medium and their relation to the developmental competence and the extent of embryonic genetic integrity. More importantly we were able to predict the apoptosis in blastocysts on E4.5, 24 h in advance i.e. on E3.5.

### Stage specific response of embryos to DNA damage

During the preimplantation development, embryos experience extensive changes in cell physiology, metabolism and chromatin architecture. Cleavage stage embryos have few DNA breaks and low transcription levels for DNA repair and cell cycle checkpoint proteins^23^. These peculiarities in cleavage stage development can influence embryonic response to genetic insults^7^. Though embryonic stem cells are believed to be extremely susceptible to DNA damaging agents^24^, preimplantation embryos respond to DNA damage in a stage specific manner^7^,^25^. Findings from this study provide evidence that the embryos carrying IDL progress normally until E2.5 and the delay is evident only on E3.5, which is in line with the findings of previous studies^7^,^26^. Developmental delay on E3.5 is likely a check point response shown by the embryos to allow enough time for the repair of DNA lesions to occur, thus preventing the genetic instability. Significant increase in the level of micronuclei in IDL embryos on E4.5 in the present study indicates that embryonic delay elicits the loss of DNA material in the form of micronuclei.

### Developmental delay and genetic instability activated apoptotic response in IDL embryos

To gain additional insights into the impact of developmental delay and genomic instability in IDL embryos, we looked at the expression of apoptotic regulators and apoptosis on E3.5 and E4.5. P53 expression in IDL embryos was approximately 0.5 fold lower than the control in E3.5 embryos and on the other hand, the expression level of P53 was increased by 1.7 fold in IDL embryo on E4.5. The insufficient function of the DNA damage checkpoint in early cleavage embryos may be caused by a low expression of genes involved prior to genomic activation^27^. In this study DNA damage checkpoint gene P53 is insufficiently transcribed, hence its transcripts were at low level on E3.5 and eventually increased at later stages, allowing a functional DNA damage checkpoint to occur. Similarly, the pro apoptotic regulator Bax also showed almost 4.5-fold increased expression in IDL embryos compared to control on E4.5 whereas anti-apoptotic Bcl-2 was poorly expressed in IDL embryos on E4.5. A major consequence of unrepaired DNA lesions is apoptosis or programmed cell death. IDL in embryos caused developmental delay and micronucleated cells which eventually resulted in cell apoptosis at the blastocyst stage possibly as a failsafe mechanism. Presence of γH2AX foci in micronuclei indicates the persistence of unrepaired DNA lesions in embryos. We used TUNEL staining as a means of detecting apoptosis on E3.5 and E4.5. In the present study, the earliest positive TUNEL signals were detected in the IDL embryos on E3.5 which were at the blastocyst stage, while no apoptosis was observed during cleavage and compaction stages. This observation is similar to the results of apoptosis assays previously reported^7^. This phenomenon is also evident in human and mouse preimplantation embryos as they do not possess an active apoptotic pathway until the post compaction phase^28^,^29^. However, it is unclear why no apoptosis is observed during the earliest stages even though DNA lesions and nuclear abnormalities were observed in embryos. Nonetheless, these observations suggest that embryos exhibit changes in the expression pattern of several apoptosis-related genes when apoptosis is activated during the development of early implantation embryos. Our results also suggest that morphologically normal embryos carrying DNA lesions exhibit increased genomic instability which may have impact on implantation and post implantation developmental periods.

### Modulation of embryonic metabolism to facilitate DNA repair

DNA repair requires an increased metabolic turnover and up regulation of metabolic pathways results in the increased uptake of nutrients from the surrounding environment^30^. This phenomenon makes it possible to understand the intrinsic state of embryos by evaluating the spent embryo culture medium non-invasively. Though, recent years have seen the development of several non-invasive methods for choosing the most suitable embryos for transfer^18^,^19^,^20^,^31^,^32^,^33^,^34^ there has been no report elucidating the significance of spent medium metabolites in the early prediction of embryonic genetic instability. Hence we looked at differential metabolic signatures between healthy embryos and embryo carrying DNA lesions. Though several energy substrates and amino acids were compared between two groups of embryos in E3.5 spent medium, only lactate, pyruvate, glucose, proline, alanine and valine levels were significantly altered in relation to the extent of genetic instability observed in E4.5 embryos. The biological significance behind this interesting observation is preliminary to conclude. Though cleavage stage embryos primarily use pyruvate, lactate and amino acids^35^, the quiet embryos hypothesis proposes that healthy embryos have lower amino acid metabolism due to lack of cellular stress^36^,^37^. Our results suggest that genetic instability in the embryos triggers the uptake of energy substrates and amino acids to facilitate the DNA repair process. Amino acids are precursors for protein and nucleotide synthesis^38^,^39^,^40^, and regulate metabolism in preimplantation embryos^41^.

### Biological significance

**Figure 8:**
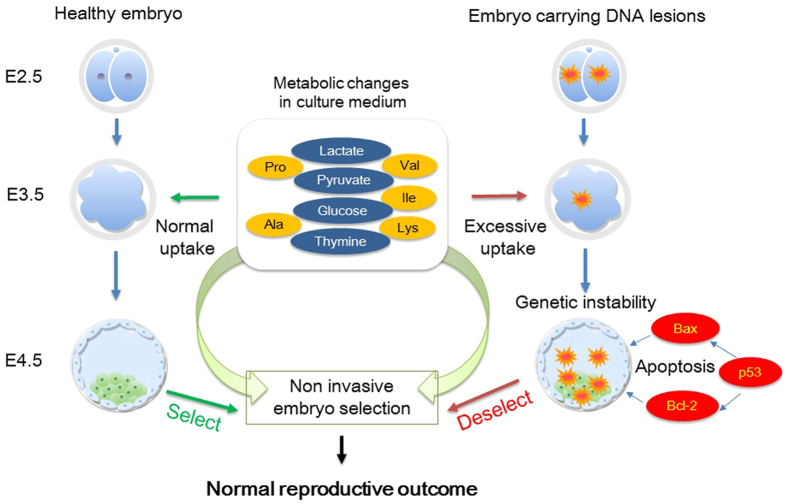
Schematic representation of non-invasive metabolomic approach in predicting DNA integrity in E4.5 embryos.

The present study reports the first metabolomics approach of embryonic response to induced DNA lesions. It provides the proof of principle that level of metabolites in embryo spent medium may allow identification of genetic integrity in embryos non-invasively. Applied to clinical setting, these biomarkers may facilitate the early prediction of genetic instability in assisted reproductive technology cycles, thereby helping in deselecting embryos with compromised genetic makeup and improve clinical outcome (Fig. 8). Further studies are necessary to determine the value of spent medium metabolites in predicting genetic instability at post-implantation developmental stages of the embryos.

## Materials and Methods

### Animals

All experiments and animal handling were conducted in accordance with the institutional guidelines for animal experimentation after obtaining prior approval from the Manipal University Institutional Animal Ethics Committee (IAEC/KMC/24/2012). Eight weeks old healthy Swiss albino male and female mice, maintained under controlled conditions of temperature (23 ± 2 °C) and light (12 h light/dark cycles) with standard diet and water ad libitum were used in this study. Experiments for each parameter were conducted in triplicate.

### Embryo collection and culture

Zygotes were retrieved from female mice primed with 5IU pregnant mare serum gonadotropin (PMSG, Cat No. G4877, Sigma Aldrich, USA) and 10IU human chorionic gonadotropin (hCG, Ovitrig, administered 48 h later) post overnight mating. Cumulus cells were removed by treatment with 0.1% hyaluronidase (Cat No. H4272, Sigma Aldrich, USA) for 30 seconds at 37 °C and cultured in KSOM AA. KSOM AA medium was prepared in-house. All components used were tissue culture grade and supplemented with essential and non-essential amino acids and 0.1% bovine serum albumin (BSA, Cat No. A9418, Sigma Aldrich, USA). Cumulus free zygotes were then visualized under phase contrast microscope (Olympus IX-71, Japan) to confirm the presence of two pronuclei (PN) and two polar bodies (PB). The zygotes were cultured in groups of 10 embryos in 20 μL of KSOM AA medium overlaid with light paraffin oil (Cat No. 61822605001730, Merck, India) at 37 °C in 5% CO_2_ in air.

### Inducing DNA lesions by *in vitro* exposure to cisplatin

Antineoplastic agent cis-Diamineplatinum(II) dichloride (Cisplatin, Cat No. P4394, Sigma Aldrich, USA) was used to induce DNA lesions in embryos. Fresh stock solution of 1 mg/ml cisplatin was prepared in phosphate-buffered saline (PBS, pH 7.4) prior to each experiment. The embryos were exposed to cisplatin at the late 2 cell stage (E1.5). The concentration and duration of cisplatin exposure was selected based on preliminary experiments conducted to determine a treatment that would induce adequate DNA damage and also allow development to blastocyst stage. Embryos were thus exposed to 3 μM cisplatin prepared in KSOM AA for 2 h at 37 °C in 5% CO_2_ in air under oil. Following exposure to cisplatin, the embryos were washed 3 times to remove all traces of the drug in Innovative Sequential Medium (ISM1, Cat No. 10500010, Origio, Denmark) and cultured in a single embryo culture system in droplets of 30 μL ISM1 at 37 °C and 5% CO_2_ under oil. For every six embryos, one media droplet without an embryo was maintained which served as an internal control henceforth referred to as medium control. The embryos were scored at 24 h intervals under phase contrast microscope (Olympus IX-71, Japan). After 48 h of culture, on E3.5, embryos were transferred to KSOM AA and the spent embryo culture media was collected after thorough mixing and individually placed into labeled 0.5 ml tubes, paraffin sealed, snap frozen in liquid nitrogen and stored at −80 °C until analysis.

### TUNEL assay

Apoptosis in embryos was assessed by Terminal deoxynucleotidyl transferase dUTP nick end labeling (TUNEL) assay kit (*In situ* Cell Death Detection Kit, TMR Red, Cat No. 12156792910, Roche, Germany). Briefly embryos were washed with PBS and placed individually onto a slide precoated with 0.01% poly L-lysine (v/v) (Cat No. P4707, Sigma Aldrich, USA) and fixed with 4% paraformaldehyde (w/v) (PFA, Cat No. P6148, Sigma Aldrich, USA) in PBS for 1 h, followed by permeabilization with 0.1% sodium citrate (w/v), 0.5% BSA (w/v) and 0.5% Triton X-100 (v/v) in PBS for 1 h at room temperature. The embryos were then washed three times in PBS with 0.5% BSA (w/v) and incubated at 37 °C for 1 h with the TUNEL reaction mixture. The embryos were then washed three times in PBS; counter stained with 4 μg/mL DAPI and observed under fluorescent microscope (Imager-A1 Carl Zeiss, Germany) for TUNEL positive cells. The embryos were evaluated for the incidence of apoptotic cells, micronuclei frequency and total cell number^29^. The labeling index was calculated as the percentage of TUNEL positive cells per blastocyst. The frequency of micronuclei was evaluated by manual counting post staining with DAPI and normalized to the cell number per embryo. MN in blastocyst were evaluated as previously described^42^,^43^. The following criteria were taken in to consideration for MN scoring. MN were considered only if they 1/16^th^ to 1/32^rd^ the size of the parent nucleus and were clearly separated from the main nucleus were counted. Presence of a nuclear envelope around MN was confirmed using correlational light electron microscopy (Fig. 5).

### Correlative Light Electron Microscopy (CLEM)

CLEM offers the benefits of combining the wide field images light (or fluorescent) microscopy with the higher resolution images of transmission electron microscopy (TEM) to spot specific cellular structures. In the present study, for the first time, CLEM is used to specifically localize the position of micronuclei stained using DAPI and imaged using confocal microscopy followed by transmission electron microscopy for high resolution imaging (Supplementary Figure S2). Briefly, the embryos were prefixed with 4% PFA and stained with 4 μg/mL DAPI, imaged on LSM 510 Meta Confocal microscope on a gridded coverslip which enables the marking of ROI (region of interest). Post fixation was performed with 1% Osmium tetroxide (OsO4) (Cat No. 75632, Sigma Aldrich, USA) and 1.5% Potassium ferrocyanide (Cat No. P3289, Sigma Aldrich, USA) solution prepared in phosphate buffer (0.1 M, pH 7.4) for 30 min at RT. Serial dehydration (30–100%) was performed with ethanol followed by infiltration and embedding with Epon-Araldite. Transmission electron microscopy was performed with a Tecnai G2 Spirit Bio-TWIN 120 KV Transmission Electron Microscope on 70 nm sections. The marked regions of the confocal images of the DAPI stained embryos were overlaid atop TEM images of the same cells collected from the serial ultrathin section. The overlay is achieved using the Adobe Photoshop version 12.0.

### γH2AX detection

Immunodetection of γH2AX was performed in cisplatin treated embryos on E4.5. Briefly, the zona free embryos were fixed with 4% PFA in PBS for 1 h and permeabilized with 0.5%Triton X 100 and 0.5% BSA in PBS for 1 h. The embryos were then treated with anti-phospho-Histone H2AX antibody (Cat No. 05636, Upstate Biotechnology, USA) at a dilution of 1:100 for 1 h at 37 °C followed by secondary antibody conjugated with FITC (goat anti-mouse IgG conjugated with fluorescein isothiocyanate, IgG-FITC, Cat No. SC-2010 Santa Cruz Biotechnology, USA) at a dilution of 1:100 for 1 h at 37 °C. The embryos were then counterstained with 4 μg/mL DAPI and observed under fluorescent microscope (Imager-A1 Carl Zeiss, Germany) at 40X magnification. Both blastomeric nuclei and MN were evaluated for the presence of γH2AX foci.

### RNA extraction and quantitative real-time PCR (qPCR)

Total RNA was extracted from approximately 30 embryos using RNAqueous micro kit (Cat No. AM1931, Ambion, Life Technologies, USA) according to the manufacturer’s instruction followed by treatment with DNAse 1. cDNA was synthesized using Protoscript First strand cDNA synthesis kit (Cat No. E6300S, New England Biolabs Inc, USA). 1 μL cDNA was used to determine the mRNA levels of P53, Bax and Bcl-2 with SYBR Green detection (Cat No. RR420, Takara, Japan) on Step One Real time PCR system (Applied Biosystems, USA). Primer sequences for qRT-PCR are provided in supplementary information (Table S1). Exon-spanning primers were used to avoid amplification of genomic DNA. Reactions were performed in triplicates in a total volume of 20 μL. The transcript levels were normalized to GAPDH. A minimum of 3 biological and 3 technical replicates were assessed.

### Metabolic profiling of spent embryo culture medium

At the time of analysis, the spent media samples were thawed to room temperature 30 min prior to sample preparation. After vortexing, 25 μL of the sample was diluted to 550 μL of 99.3 μM Sodium salt of 2,2,3,3 tetradeutero 3-(trimethyl silyl) propionate (TSP, Cat No. 269913, Sigma Aldrich, USA) in ^2^H_2_O and transferred to a 5 mm NMR tube for acquisition. TSP was used as a standard reference molecule.

All spectra were acquired at 298 K on a Bruker AVANCE NMR spectrometer operating at a ^1^H resonance frequency of 800 MHz equipped with a cryogenically cooled triple resonance (^1^H, ^13^C, ^15^N) probe. Signals from the protein component of the media were suppressed using a Carr- Purcell-Meiboom-Gill (CPMG) sequence of 20 ms delay, which was incorporated into the one- dimensional (1D) radio-frequency pulse scheme. Suppression of the water (H_2_O) was achieved with pre-saturation (r.f. strength = 50 Hz). The 1D ^1^H NMR spectra was acquired using a ^1^H 900 pulse width of 8 μs, relaxation delay (trel) of 7 s between scans, spectral width of 9600 Hz and an acquisition time (tmax) of 1.1 s (16 K complex points). The total of trel + tmax ~ 8 seconds was used to ensure that it exceeds 3*T1 of the metabolites being studied. A total of 400 transients were collected resulting in a measurement time of 53 min for each sample. The time domain data was apodized with an exponential window function (line broadening 0.3 Hz) and zero-filled to 64 K points prior to Fourier transform.

### NMR data analysis

The NMR spectrum was analyzed using the Bruker TOPSPIN 3.2 software. The peaks of specific metabolites were confirmed with spiking experiments and other new NMR methods as described previously^44^. The metabolite peaks were measured with respect to the TSP signal (which was normalized to 1.0). The NMR data is therefore represented as peak intensities thus have no units.

### Statistical Analysis

All data analysis was performed using GraphPad Instat Software Inc., USA. Blastocyst rate and hatching rate is represented as percentage data and was evaluated by Chi Square statistics. The different variables are expressed as Mean ± SEM (Standard error of mean). Student’s t-test was applied to normally distributed data and Mann-Whitney U-test was applied to data that did not conform to normal distribution. P value < 0.05 was considered statistically significant. The graphs were plotted using Microcal Origin 6.0 (USA).

## Additional Information

**How to cite this article**: D’Souza, F. *et al.* Unraveling the association between genetic integrity and metabolic activity in pre-implantation stage embryos. *Sci. Rep.* **6**, 37291; doi: 10.1038/srep37291 (2016).

**Publisher’s note**: Springer Nature remains neutral with regard to jurisdictional claims in published maps and institutional affiliations.

## Supplementary Material

Supplementary Information

Supplementary Information

## Figures and Tables

**Table 1:** Intensities of metabolites from spent culture medium on E3.5 in relation to developmental stage on E3.5.

	Control		IDL	
	Normally Developed N = 33	Delayed N = 39	Normally Developed N = 34	Delayed N = 63
Lactate	0.183 ± 0.011	0.209 ± 0.008	0.202 ± 0.009	0.194 ± 0.009
Glucose	0.021 ± 0.002	0.027 ± 0.002^*^	0.021 ± 0.001	0.023 ± 0.001
Pyruvate	0.170 ± 0.021	0.180 ± 0.008^**^	0.202 ± 0.027	0.173 ± 0.018
Thymine	0.061 ± 0.004	0.080 ± 0.004^**^	0.061 ± 0.003	0.071 ± 0.003^*^
Proline	0.026 ± 0.002	0.029 ± 0.002	0.029 ± 0.004	0.032 ± 0.002
Lysine	0.044 ± 0.007	0.051 ± 0.005	0.039 ± 0.004	0.037 ± 0.006
Alanine	0.032 ± 0.002	0.040 ± 0.002^**^	0.037 ± 0.002	0.039 ± 0.002
Valine	0.072 ± 0.004	0.086 ± 0.004^*^	0.08 ± 0.004	0.078 ± 0.003
Isoleucine	0.033 ± 0.002	0.039 ± 0.001^*^	0.036 ± 0.002	0.037 ± 0.002

*p < 0.05, **p < 0.01 Vs corresponding normally developed group.

**Table 2:** Intensities of metabolites from spent culture medium on E3.5 in relation to developmental stage on E4.5.

	Control		IDL	
	Normally Developed N = 51	Delayed N = 11	Normally Developed N = 72	Delayed N = 13
Lactate	0.184 ± 0.008	0.217 ± 0.017	0.191 ± 0.008	0.185 ± 0.018
Glucose	0.024 ± 0.002	0.029 ± 0.003	0.0219 ± 0.001	0.024 ± 0.003
Pyruvate	0.161 ± 0.015^a^	0.186 ± 0.016^**^	0.183 ± 0.019	0.152 ± 0.019
Thymine	0.062 ± 0.003^b^	0.088 ± 0.01^*^	0.063 ± 0.002	0.076 ± 0.006
Proline	0.024 ± 0.002	0.029 ± 0.004	0.029 ± 0.002	0.038 ± 0.006
Lysine	0.047 ± 0.006	0.051 ± 0.006	0.041 ± 0.006	0.032 ± 0.007
Alanine	0.034 ± 0.002	0.041 ± 0.004	0.038 ± 0.002	0.036 ± 0.003
Valine	0.07 ± 0.003^b^	0.087 ± 0.008^*^	0.074 ± 0.003	0.075 ± 0.007
Isoleucine	0.032 ± 0.001	0.038 ± 0.003	0.036 ± 0.002	0.034 ± 0.004

*p < 0.05, **p < 0.01 Vs corresponding normally developed group.
