# Supplementary material for: Unraveling the association between genetic integrity and metabolic activity in pre-implantation stage embryos
Source: Sci Rep. 2016 Nov 17;6:37291. doi: 10.1038/srep37291 (PMC5112559; doi:10.1038/srep37291)
Supplement: Supplementary Information [file srep37291-s1.doc]

**Title:** Unraveling the association between genetic integrity and metabolic activity in pre-implantation stage embryos.

**Author affiliation:** Fiona D’Souza^1^, Shivananda M. Pudakalakatti^2,3^, Shubhashree Uppangala^1^, Sachin Honguntikar^1^, Sujith Raj Salian^1^, Guruprasad Kalthur^1^, Renu Pasricha^4^, Divya Appajigowda^4^, Hanudatta S. Atreya^2^, Satish Kumar Adiga^1^

^1^Division of Clinical Embryology, Centre of Excellence in Clinical Embryology, Kasturba Medical College, Manipal University, Manipal. ^2^NMR Research Centre and ^3^Solid State and Structural Chemistry Unit, Indian Institute of Science, Bangalore, India. ^4^National Centre For Biological Sciences, TIFR, Bengaluru, India

**Corresponding authors:**

**Satish Kumar Adiga, Ph.D**.

Division of Clinical Embryology, Centre of Excellence in Clinical Embryology, Kasturba Medical College, Manipal University, Manipal-576 104, India Tel: 91-820-29-22320,

E mail: [satish.adiga@manipal.edu](mailto:satish.adiga@manipal.edu)

**Hanudatta S. Atreya, Ph.D**

NMR Research Centre, Indian Institute of Science, Bangalore-560012, India

Email: [hsatreya@sif.iisc.ernet.in](mailto:hsatreya@sif.iisc.ernet.in)

Correlative Light Electron Microscopy (CLEM)


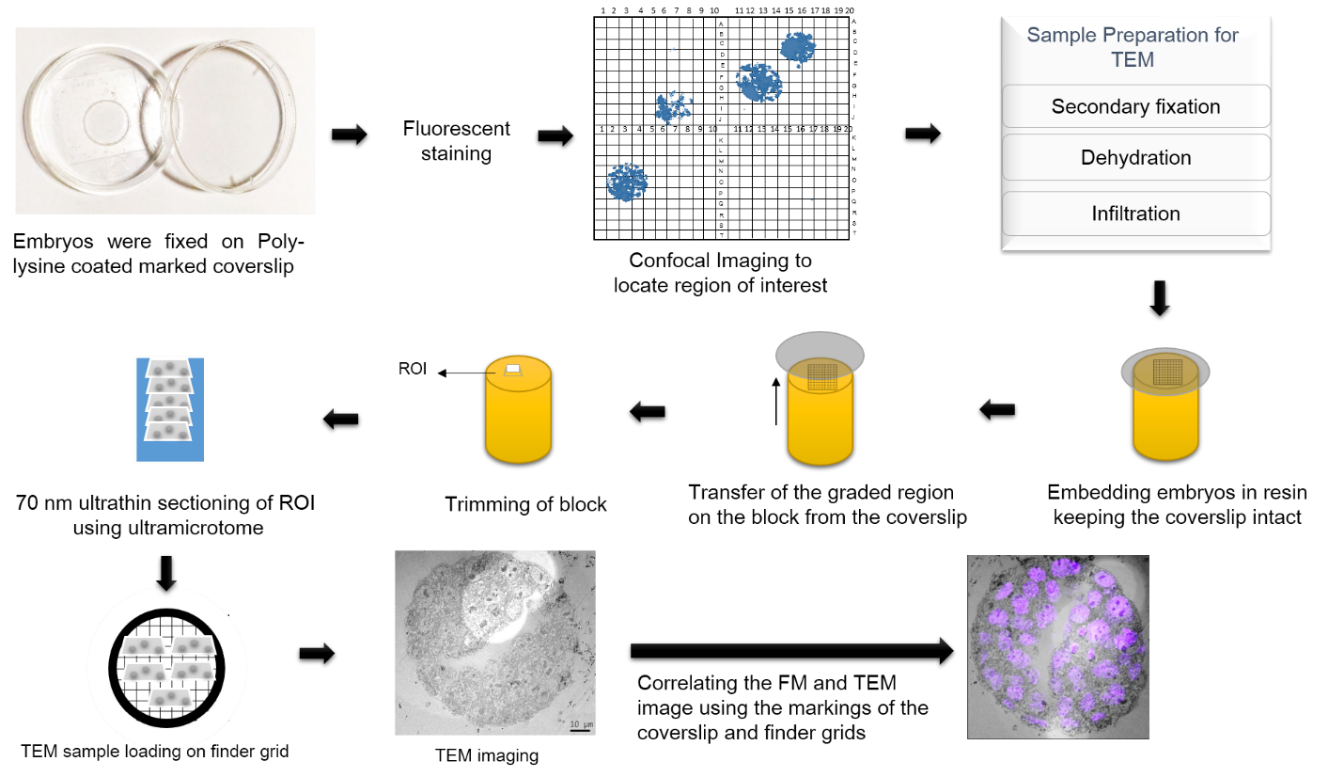


Fig S2 Correlative Light Electron Microscopy (CLEM)

The major drawback of fluorescence microscopy is that it lacks information on the ultrastructural levels. Transmission Electron Microscopy (TEM) is the most sensitive tool available to achieve high resolution ultrastructural visualization. The challenge with TEM is the identification of the multitude of subcellular structures. Reports have shown that TEM has been applied for the detection of micronuclei (Rello-Varona et al., Cell cycle 2012, Schiffmann et al ., Mutation Research1991.

In order to overcome the limitations of fluorescence microscopy based detection of micronuclei, we attempted a novel approach; CLEM. CLEM provides a unique platform to combine the authenticity and higher resolution images of transmission electron microscopy (TEM) with the power of the wide field images light (or fluorescent) microscopy, thereby facilitating rapid identification of specific cellular structures and ultrastructural changes. CLEM has been used in the identification and localization of cellular and subcellular events. (de Boer et al., Nature methods 2015, Polishchuk et alThe Journal of cell biology 2000., Olmos et al ., Nature 2015.

In the present study, CLEM is used to specifically localize the position of micronuclei (Supplementary figure S2). Blastocysts were fixed on a gridded coverslip which enables the marking of ROI (region of interest) with 4% PFA followed by nuclear staining using 4µg/mL DAPI. Fluorescence imaging was carried out at 10X, 20X and 63X magnification on LSM 510 Meta Confocal microscope. This was followed by post fixation for Transmission electron microscopy which was performed with 1% Osmium tetroxide (OsO_4_) (Cat No. 75632, Sigma Aldrich, USA) and 1.5% Potassium hexacyanoferrate(II) trihydrate (K_4_Fe(CN)_6_.3H_2_O) (Cat No. P3289, Sigma Aldrich, USA) solution prepared in phosphate buffer (0.1M, pH 7.4) for 30min at RT. Serial dehydration (30% - 100%) of the embryos was performed with ethanol followed by infiltration and embedding with Epon-Araldite. To embed the embryos onto Epon-Araldite, a capsule was filled with Epon-Araldite which was placed directly on the ROI and the resin was allowed to polymerize at 60˚C for 48h. The resin block was then sectioned on a microtome to make 70nm serial ultrathin sections which were loaded on grid and allow to dry. Transmission electron microscopy was performed with a Tecnai G2 Spirit Bio-TWIN 120 KV Transmission Electron Microscope. The marked regions of the confocal images of the DAPI stained embryos were overlaid atop TEM images of the same cells collected from the serial ultrathin section. The overlay was achieved using the Adobe Photoshop version 12.0. Micronuclei visualized with DAPI staining were confirmed by ascertaining the presence of a nuclear envelope (Schiffmann et al., Mutation Research 1991).
