# Supplementary material for: Unraveling the association between genetic integrity and metabolic activity in pre-implantation stage embryos
Source: Sci Rep. 2016 Nov 17;6:37291. doi: 10.1038/srep37291 (PMC5112559; doi:10.1038/srep37291)
Supplement: Supplementary Information [file srep37291-s2.doc]

**Title:** Unraveling the association between genetic integrity and metabolic activity in pre-implantation stage embryos.

**Author affiliation:** Fiona D’Souza^1^, Shivananda M. Pudakalakatti^2,3^, Shubhashree Uppangala^1^, Sachin Honguntikar^1^, Sujith Raj Salian^1^, Guruprasad Kalthur^1^, Renu Pasricha^4^, Divya Appajigowda^4^, Hanudatta S. Atreya^2^, Satish Kumar Adiga^1^

^1^Division of Clinical Embryology, Centre of Excellence in Clinical Embryology, Kasturba Medical College, Manipal University, Manipal. ^2^NMR Research Centre and ^3^Solid State and Structural Chemistry Unit, Indian Institute of Science, Bangalore, India. ^4^National Centre For Biological Sciences, TIFR, Bengaluru, India

**Corresponding authors:**

**Satish Kumar Adiga, Ph.D**.

Division of Clinical Embryology, Centre of Excellence in Clinical Embryology, Kasturba Medical College, Manipal University, Manipal-576 104, India Tel: 91-820-29-22320,

E mail: [satish.adiga@manipal.edu](mailto:satish.adiga@manipal.edu)

**Hanudatta S. Atreya, Ph.D**

NMR Research Centre, Indian Institute of Science, Bangalore-560012, India

Email: [hsatreya@sif.iisc.ernet.in](mailto:hsatreya@sif.iisc.ernet.in)


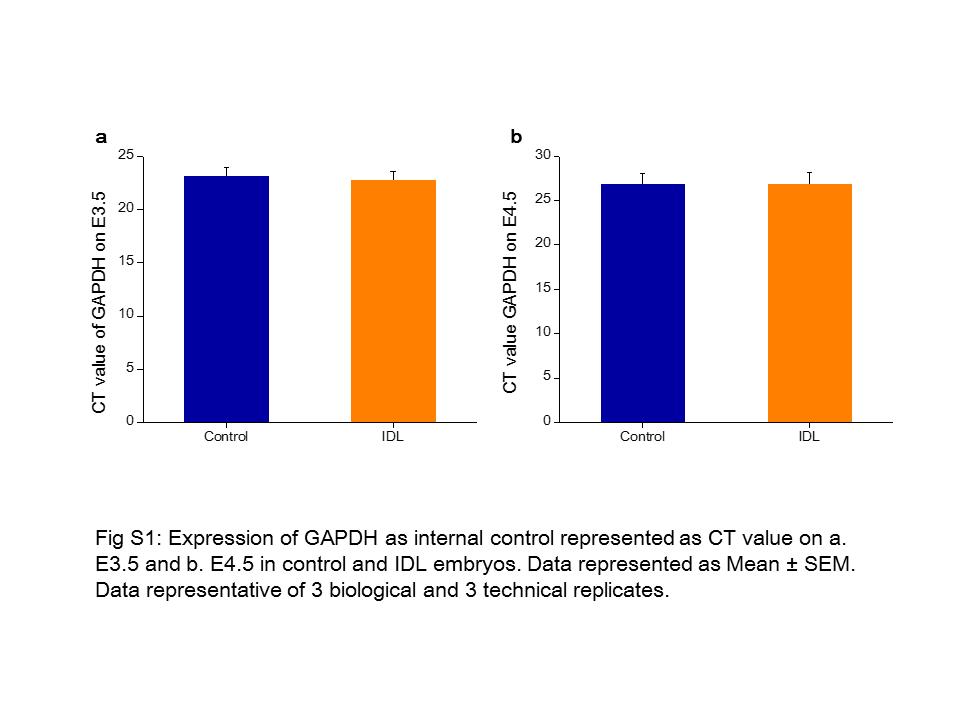


Table S1: Intensities of metabolites from spent culture medium on E3.5 normalized to cell number

|  | Control | IDL |
| --- | --- | --- |
| Lactate | 0.0082 + 0.0006 | 0.0080 + 0.0007 |
| Glucose | 0.0008 + 0.0001 | 0.0008 + 0.0001 |
| Pyruvate | 0.0076 + 0.0005 | 0.0072 + 0.0006 |
| Thymine | 0.0031 + 0.0002 | 0.0027 + 0.0002 |
| Proline | 0.0012+ 0.0002 | 0.0009 + 0.0001 |
| Lysine | 0.0014 + 0.0001 | 0.0014 + 0.0002 |
| Alanine | 0.0014 + 0.0001 | 0.0014 + 0.0001 |
| Valine | 0.0037 + 0.0002 | 0.0035 + 0.0003 |
| Isoleucine | 0.0015 + 0.0001 | 0.0015 + 0.0001 |

Please note that no significant differences were observed between the groups when metabolism was normalized to cell number.

Table S2. Primer sequences for qRT-PCR

| **Gene** | **Forward Primer** | **Reverse Primer** |
| --- | --- | --- |
| P53 | 5’-GACCGCCGTACAGAAGAAGA-3’ | 5’-GCGGATCTTGAGGGTGAAATA-3’ |
| Bax | 5’- ATCTGGTTCTGCAAGCGTTTA-3’ | 5’-CCTGCTCCGAATTTGGTGAAA-3’ |
| Bcl-2 | 5’- ATGCCTTTGTGGAACTATATGGC- 3’ | 5’-GGTATGCACCCAGAGTGATGC-3’ |
| GAPDH | 5’- AGGTCGGTGTGAACGGATTTG- 3’ | 5’-TGTAGACCATGTAGTTGAGGTCA-3’ |
